# Bio-efficacy of *Syzygium aromaticum* bud extract as eco-friendly green acaricide for controlling *Hyalomma dromedarii*

**DOI:** 10.1038/s41598-026-58848-6

**Published:** 2026-07-01

**Authors:** Salma Nabil Ahmed Mohamed, Ashraf Ahmed Montasser, Ahmed Mohamed Hamdy Nigm, Asmaa Ali Baioumy Ali

**Affiliations:** Zoology Department, Faculty of Science, Ain Shams University, Abbassia, Cairo, 11566 Egypt

**Keywords:** Bioactivity, Clove, Extraction, Ticks, Phytochemicals, Protein, Biochemistry, Drug discovery, Plant sciences, Zoology

## Abstract

The most common hard tick in Egypt is *Hyalomma dromedarii* as a one of the most dangerous ectoparasites. The current study aims to detect the chemical composition of *Syzygium aromaticum* bud ethanolic extract using GC-MS and some phytochemical analysis in addition to investigating its acaricidal activity on *H*. *dromedarii* engorged females through biological and biochemical studies. The GC-MS revealed the presence of eugenol, eugenol acetate and D-(-)-fructofuranose as the most active compounds, and it appeared to be rich in alkaloids, flavonoids, phenolic compounds, tannins and terpenoids. At high concentration, the extract caused mortality to reach 100% after 35 days of treatment in engorged females, with LC_50_ reached 100.22 and 0.83 mg/mL after the 3rd and 5th week (half and end of examined period) following treatment, respectively. The blood digestion was affected by significantly decreasing in their body weights. Furthermore, the extract showed a decrease in their oviposition, hatchability, and fertility. The extract at 100 mg/mL caused a reduction in ovary total protein content with disappearance of some protein bands using SDS-PAGE, compared with the control group. Overall, the current study revealed the effectiveness of *S*. *aromaticum* extract as a bio-acaricide for controlling *H*. *dromedarii* and it recommended numerous additional field studies to assess its effectiveness under different conditions.

## Background

Globally, arthropods such as ticks act as vectors of many human and animal pathogens including viruses, bacteria, and protozoa^1^. *Hyalomma* tick species pose major threats to camels and other livestock across Africa, Eastern Europe, the Middle East, and Western Asia^2^. In Egypt, *Hyalomma dromedarii* has been reported to infest livestock, both domestic and imported animals for the Egyptian market, including buffaloes, camels, sheep, cattle and goats, as well as dogs and migratory birds crossing the country^3,4^. In addition, 76.44% of the examined camels were infested with ticks, with a prevalence of 17% of *Hyalomma dromedarii*^5^. They caused tick-borne diseases (TBDs) such as babesiosis, theileriosis, anaplasmosis, rickettsiosis, ehrlichiosis, and borreliosis which have been detected in different governorates of Egypt^3,6^.

*Hyalomma dromedarii* affects the epidemiology of Dera Ghazi Khan virus^7^, Quaranfil virus^8^, Chick Ross virus, Kadam virus and Sindbis virus^9^, Bhanja virus^10^, spotted fever rickettsia (*Rickettsia rickettsii*)^11^, Thogoto virus^12^, African tick-borne fever (*Rickettsia africae*)^13^, Q fever (*Coxiella burnetii*)^14^, theileriosis of cattle (*Theileria annulata*)^15^, theileriosis of camels (*T*. *camelensis*)^16^, and Crimean-Congo hemorrhagic fever virus^17^. So its control is critical for the prevention of tick-borne diseases^18,19^.

Tick infestations are recognized as an increasing problem in the camel industry^20^. The efficacy of acaricides is not well characterized for tick control although a wide variety of people use them. Even though chemical acaricides are still thought to be the most effective method of control, they have a number of drawbacks, such as high costs, hazardous effects, lengthy wait times, and marketed acaricides’ chemoresistance^21^. The search for new substitutes and the need for safer products with novel mode of action and less toxicity to both humans and the environment are critically needed. Currently, there is a lot of interest in botanical alternatives, such as essential oils and plant extracts^22^. Nowadays, medicinal plants are being given more attention because of their phytoconstituents’ pharmacological effectiveness, fewer adverse effects, and higher survivability when compared to their synthetic counterparts.

The genus *Syzygium* is one of the genera of the myrtle family, Myrtaceae^23^. Many species of this genus are known for their traditional use in various diseases^24^. Cloves are rich in many phytochemicals, including terpenoids, phenolic compounds, carbohydrates, glycosides, and alkaloids^25,26^. A review of previous reports suggests that clove possesses biologically active components that are effective against many types of diseases^27–33^. Its bioactive compounds exhibited antioxidant activity^34^, antiviral activity^35^, and anticandidal and antiproliferative activities^36^. It is rich in eugenol, a compound well-documented for its anti-parasitic activity^37–39^. This property has been widely studied, showing that it effectively reduces parasites load and disrupts their life cycle^40,41^.

The current study aimed to characterize the phytochemicals that are present in the *S*. *aromaticum* extract, especially the ethanolic ones, and to evaluate its acaricidal activity at different concentrations against *H*. *dromedarii* through the main biological parameters that affect their development and reproduction, in addition to biochemical studies through ovary protein contents.

## Methods

### Plant collection

The flower buds of the clove plant, *Syzygium aromaticum*, used in this study were bought dried from a local market in Egypt. They were identified by a botanist at the Botany Department, Faculty of Science, Ain Shams University. Buds were cleaned from any dust using a small painting brush, then ground using a stainless-steel knife mill (Braun Multiquick Chopper System, B098TZFGHM).

### Plant extract preparation and analysis

#### Ethanolic extract preparation

The ethanolic extract of clove was prepared according to Alimi et al. ^22^. It was prepared by adding 100 g of plant powder to 500 mL of 80% ethyl alcohol, covered with aluminum foil and kept in dark condition for 72 h at room temperature. The mixture was filtered using filter paper (Whatman No. 1, 110 mm). The filtrate was poured into glass petri dishes and put in the incubator at 60 ˚C for alcohol evaporation. Finally, the dried extract was collected, weighed, transferred to clean glass vials and kept at 4 ˚C until use.

A stock solution of 400 mg/mL was prepared by dissolving 4 g in 10 mL of 2% Tween 80 (2 mL Tween 80 in 100 mL distilled water). Serial dilutions were made from this concentration to prepare concentrations of 200, 100, 50 and 25 mg/mL.

#### Analysis of Syzygium aromaticum Extract

##### Extraction yield

The extract yield was calculated according to the following equation: Extract yield %= A/B X 100, where A is the weight of the dried ethanolic clove bud extract and B is the weight of the raw ground clove bud powder^42^.

##### Gas chromatography-mass spectrometry (GC-MS)

Chromatographic analysis of the extract was performed using GC-MS (Agilent Technologies 7890B GC Systems combined with 5977 A Mass Selective Detector). The sample was analyzed with the capillary column (HP-5MS Capillary; 30.0 m × 0.25 mm ID × 0.25 μm film) held initially for 5 min at 60 ˚C after 1 µL injection using helium as a carrier gas at a pressure of 8.2 psi. Then, the temperature was increased to 300 ˚C with a 20 ˚C/min heating ramp, with a 5.0 min hold. Injection was carried out in split mode (10:1) at 300 ˚C. MS scan range was (*m*/*z*): 50–550 atomic mass units (AMU) under electron impact (EI) ionization (70 eV) and solvent delay 8.0 min.

The reaction is carried out by adding 300 µL of Silylation agent BSTFA (N, O- bis (trimethylsilyl) trifluoroacetamide) to amount of the sample after extraction and heating in water bath at 80 ˚C for two hrs and after that injected into GC-MS under the above conditions. The constituents were determined by mass fragmentations with The NIST mass spectral search program for the NIST/EPA/NIH mass spectral library Version 2.2 (Jun 2014).

#### Phytochemical analysis

##### Total alkaloids

The plant powder samples were defatted using petroleum ether; ethyl alcohol was added to 10 g of defatted dried powder and refluxed for two days, then the solution was filtered. The filtrate was concentrated in a water bath at 70 ^◦^C until complete dryness, then a few drops of H_2_O and 3 drops of conc. HCl were added. The solution was transferred to a separating funnel, and chloroform was added. The lower layer was discarded, and chloroform was added to the remaining layer. The last step was repeated three times. The remaining layer was transferred to a clean weighed beaker and adjusted to 7.5-8 pH. The beaker was put in a water bath at 70 ^◦^C until complete dryness, and finally the beaker was weighed^43^.

The concentration was calculated according to the following equations: Residue = weight of beaker with residue – weight of the empty beaker. Concentration (mg/g) = weight of residue / weight of plant.

##### Total flavonoids

Total flavonoid contents (TFC) were determined by the aluminum chloride (AlCl_3_) colorimetric method^44^. The plant dried samples were defatted using petroleum ether; ethyl alcohol was added to 2 g of defatted dried powder, and then the solution was filtered. The filtrate was concentrated in a water bath till it became less than 50 mL; the volume was adjusted to 50 mL using ethyl alcohol (stock solution). A 0.5 mL sample was taken in a test tube and put in a water bath till complete dryness. Finally, 5 mL of 0.1 M AlCl_3_ (1.334 g in 100 mL methanol) was added to the dried powder and mixed well.

The absorbance was measured at 445 nm using a UV-Vis spectrophotometer. The total flavonoid content was estimated using a quercetin standard curve, and the results are expressed as mg quercetin equivalents. The concentration was calculated according to the following equations: X (ppm) = 0.998 − 0.004 Y (absorbance) / 0.096. mg/g = ppm * dilution (50 mL) / weight (2 g) * 10,000 * 0.5 mL.

##### Total phenolic compounds

According to Snell and Snell^45^, ten drops of concentrated hydrochloric acid were added to 200 µL of sample extract (200 µL of distilled H_2_O was used as a blank). The solution was heated rapidly to boiling point over a flame, then it was placed in a boiling water bath for 10 min. and was covered with foil. After cooling, 1 mL of Folin reagent and 5 mL of 20% Na_2_CO_3_ solution were added. The mixture was completed to 10 mL with distilled water. The absorbance was measured at 520 nm after 30 min of incubation at room temperature. The standard of gallic acid was used as the calibration curve. The results are expressed in mg gallic acid equivalents per gram extract (mg GAE/g extract).

##### Total tannin

Two grams of dried plant powder were added to 100 mL H_2_O in a flask and refluxed for 1 h. The solution was filtrated and 30 mL of 5% copper acetate was added. The solution was boiled for 10 min. with stirring, and then it was kept at room temperature for 24 h for precipitation. Then it was filtrated with ashless filter paper and washed with water. The ashless filter paper was put in the previously weighed crucible, then ignited at 600 ^◦^C for one hr, and finally, the crucible was weighed after ignition.

The concentration was calculated according to the following equations: Residue = weight of crucible with residue – weight of the empty crucible, concentration (mg/g) = weight of residue / weight of plant^46^.

##### Total terpenoids

Total terpenoid contents (TTC) were determined by soaking 100 mg (wi) of dried plant extract in 9 mL of ethanol for 24 h. The solution was filtered and 10 mL of petroleum ether was added for extraction using separation. The ether extract was separated into pre-weighed glass vials and waited for its complete drying (wf). Ether was evaporated, and the yield of total terpenoid contents was measured by the formula: Concentration (mg/g) = wi – wf /wi^47^.

### Tick collection

Naturally infested camels were used to collect the camel tick, *Hyalomma dromedarii* (Ixodoidea: Ixodidae) at the Birqash camel market (30˚ 9‵ 58.4‶ N, 31˚ 2‵ 13.2‶ E), Giza Governorate, Egypt. The ticks were collected from the animal immediately after feeding, once the mouthparts had detached from the skin, also, if they dropped to the ground, the collection performed from there immediately. According to Walker^48^, the collected ticks were identified and grouped into non-engorged, semi-engorged, and engorged adults. Only engorged females were used in the present study. They were kept in glass vials covered by pieces of gauze in the incubator at 28 ± 2˚C and 75–80% relative humidity (RH) for adult experiments preparation within 24 h of collection.

### Tick treatment

#### Bioassay protocols: testing acaricidal activity

Adult engorged female *H. dromedarii* were used to study some biological parameters that affect development and reproduction. According to Drummond et al.^49^, the adult immersion test (AIT) was performed. The engorged female ticks were weighed and assigned to groups of 20 ticks in each one. Each group was separately immersed in 10 mL of each concentration of *S. aromaticum* ethanolic extract (25, 50, 100, 200 or 400 mg/mL) for five min.

Ticks were then transferred to filter papers for drying and kept separately in glass vials that were closed by gauze. Three replicates were made for each concentration, each trial contained about seven females. The treated ticks were kept in the incubator at 28 ± 2˚C and 75–80% RH to provide optimum conditions for oviposition. Females were observed for duration of five weeks following feeding and treatment. The groups that received the plant extract at different concentrations were referred to herein as treated ones. The comparison was done between treated groups and control ones that received water and 2% Tween 80^50–53^.

##### External morphological studies

Changes in the morphology of tick body were visualized using a stereomicroscope (Olympus^®^ Binocular Upright Microscope, Hamburg, Germany). The morphological appearance of control groups was compared with those of treated ones at various concentrations. Females were photographed using a Samsung ES95 HD digital camera.

##### Mobility and viability

Every two days during the successive five weeks following treatment, mobile, immobile, and dead ticks were examined. To detect their movement, the individual females were pressed several times on their dorsal side by the soft forceps. Viability was determined by the percentage of live and dead ones. The death of ticks was assessed by darkening in their integument^54^. Also, they were considered dead if they did not respond when their legs were forcibly stretched^55^. The percentages of ticks in each case were calculated by the following: number of ticks (mobile or immobile or dead)/total number of ticks X 100.

After the 1st, 2nd, 3rd, 4th, and 5th weeks following treatment, lethal concentration (LC) of the clove extract was detected as LC_50_ (lethal concentration at which the mortality rate reached 50%).

The following equation (%E = B - T/B × 100), where B is the mean number of surviving ticks in the control group and T is the mean number of surviving ticks in the treated group^18,56^ was used to determine the acaricide efficacy of *S. aromaticum* ethanolic extract at different concentrations after each examined week of treatment.

##### Blood digestion monitoring

Blood digestion was detected by weighing fully engorged females directly after feeding (zero week) and during the successive five weeks following feeding and treatment. It was calculated by subtracting the tick weight of each week from the weight of the week before. The weight of laid eggs was added to the weight of their females in each week^18^.

##### Reproductive metrics: oviposition, hatchability and fertility

After oviposition, the eggs produced by each female were collected, weighed, and kept in separate glass vials covered by gauze at the incubator under optimum conditions for hatching.

Oviposition was represented by oviposition percent (the number of oviposited females/total number of females × 100), pre-oviposition period (days from engorgement to onset of egg laying), and number of eggs per ovipositing female. Hatchability was represented by the hatching period (number of days starting from onset of egg laying to their hatch) and the hatching percent (number of hatched eggs/ total number of eggs × 100). Fertility is the weight of egg mass divided by the weight of a replete female, and the weight of one egg is the weight of egg mass divided by the total number of eggs^18^.

The effect of the extract on the fecundity was detected by the calculation of the Reproductive Index (RI) (the average weight of laid eggs (mg)/ average weight of female ticks (mg)), then the inhibition of oviposition (IO%) was determined by the following equation ((RI control - RI treated)/ RI control) × 100). The reproductive efficiency (RE) was detected by egg weight x hatching percent x 20.000 */ weight of females, (* constant indicating the number of eggs present in 1 g of egg laying). Effectiveness of the product (PE) = RE (control group) - RE (treated group) / RE (control group) × 100 ^53^.

#### Biochemical studies on ovarian tissues (protein analysis)

##### Preparation of ovary samples

Fully fed female *H*. *dromedarii*, both untreated and treated with 100 mg/mL of *S*. *aromaticum* ethanolic extract were dissected after one week of feeding and treatment (15 engorged females divided into three replicates 5 per each one) for the treated group and the same for the control one. Then, Ovaries were collected, weighed and frozen at -80 ˚C until samples preparation. The ovaries were pooled and homogenized in cold phosphate buffer (1:3 w/v). They were centrifuged at 3000 rpm for 20 min. and the supernatant was collected and stored at -80 ˚C until analyzed.

##### Quantitative analysis

A colorimetric Biuret method of the Biodiagnostic total protein kit was used to detect the total protein concentration of the samples^57^. In the total protein kit, 25 µL of the standard protein reagent (5 g/dL) was added to 1 mL of the Biuret reagent (6 mmol/L cupric sulfate, 21 mmol/L sodium potassium tartrate, 750 mmol/L sodium hydroxide, and 6 mmol/L potassium iodide) for standard preparation. For sample preparation, 25 µL of the sample supernatant was added to 1 mL of the Biuret reagent. They were incubated for 10 min. at 37 ˚C. The spectrophotometer was used to measure the absorbance of standard and samples at 550 nm. The protein concentration was calculated according to the following equation: Conc. (g/dL) = Absorbance of sample / Absorbance of standard * 5.

##### Qualitative analysis using sodium dodecyl sulfate polyacrylamide gel electrophoresis (SDS-PAGE)

The most widely used system for electrophoresis of proteins is probably that described by Laemmli et al.^58^. It is a discontinuous system for resolving proteins denatured with SDS.

A 1.5 mm thick gel consisted of the separating gel, pH 8.8 (3.3 mL monomer solution (30% T, 2.5% C Bis), 2.5 mL 1.5 M Tris-HCl, pH 8.8, 0.1 mL 10% SDS, 75 µL 10% ammonium persulphate (APS), 10 µL TEMED, and 4 mL distilled H_2_O) and the stacking gel, pH 6.8 (0.44 mL monomer solution (30% T, 2.5% C Bis), 0.83 mL 0.5 M Tris-HCl, pH 6.8, 30 µL 10% SDS, 75 µL APS, 10 µL TEMED, and 2 mL distilled H_2_O) were poured in a vertical slab gel unit (Vertical Electrophoresis Cell Apparatus Modular Dual Vertical Electrophoresis DYCZ-24DH). To prepare the sample for electrophoresis, the sample was mixed with an equal volume of the sample buffer (1.25 mL 0.5 M Tris-HCl, pH 6.8, 2 mL 10% SDS, 1 mL glycerol, 0.5 mL mercaptoethanol, drops of bromophenol blue stain, and 5 mL distilled H_2_O) and put in boiling water for about 90 s. Then the treated sample was stored frozen until using it. 20 µL of each sample was loaded into the well under the electrode buffer using the gel loading tip of electrophoresis. A standard protein marker with a broad range of molecular weights 5-245 kDa, was used (BLUelf Prestained Protein Ladder, GeneDireX). A power supply was used to conduct the electric current to the device. The run was carried out at 100 V for about 2 h until the tracking dye reached the end of the resolving gel. Coomassie Brilliant Blue stain (CBB) was used to stain the gel. The staining gel was left on the shaker overnight at room temperature. For band appearance, the gel was shaken for about 1 h in destaining solution I and for about 6 h in destaining solution II. The gel was washed with distilled water and put in the fixing solution (glycerol and distilled water at a ratio of 1:1). Gel was observed and photographed using a Samsung ES95 HD digital camera.

### Statistical analysis

Each experiment was conducted in triplicate and the results represent the mean of these three replicates. Most data are presented as mean ± SE. Statistical analyses were conducted by GraphPad Prism V9.5.1 (GraphPad^®^ Software Inc., San Diego, CA, USA). The one-way ANOVA test followed by Tukey’s multiple comparisons test was applied for multiple groups^41^. Differences were considered significant at *P* < 0.05, highly significant at *P* < 0.01, and very highly significant at *P* < 0.001 and *P* < 0.0001. Lethal concentrations (LC) were estimated using Probit analysis (Probit^®^ Probit Vb6)^59^.

The protocol and procedures were approved by the Research Ethics Committee of Ain Shams University (RECASU), Faculty of Science, Ain Shams University, Code: ASU-SCI/ZOOL/2025/1/4.

## Results

### Plant extract analysis

#### Extraction yield

The ethanolic extraction of clove bud resulted in 10.6% of the soluble materials dissolved in ethanol, according to the applied operating mode and dry matter weight calculation (w/w).

#### Gas chromatography-mass spectrometry (GC-MS) analysis

**Fig. 1:**
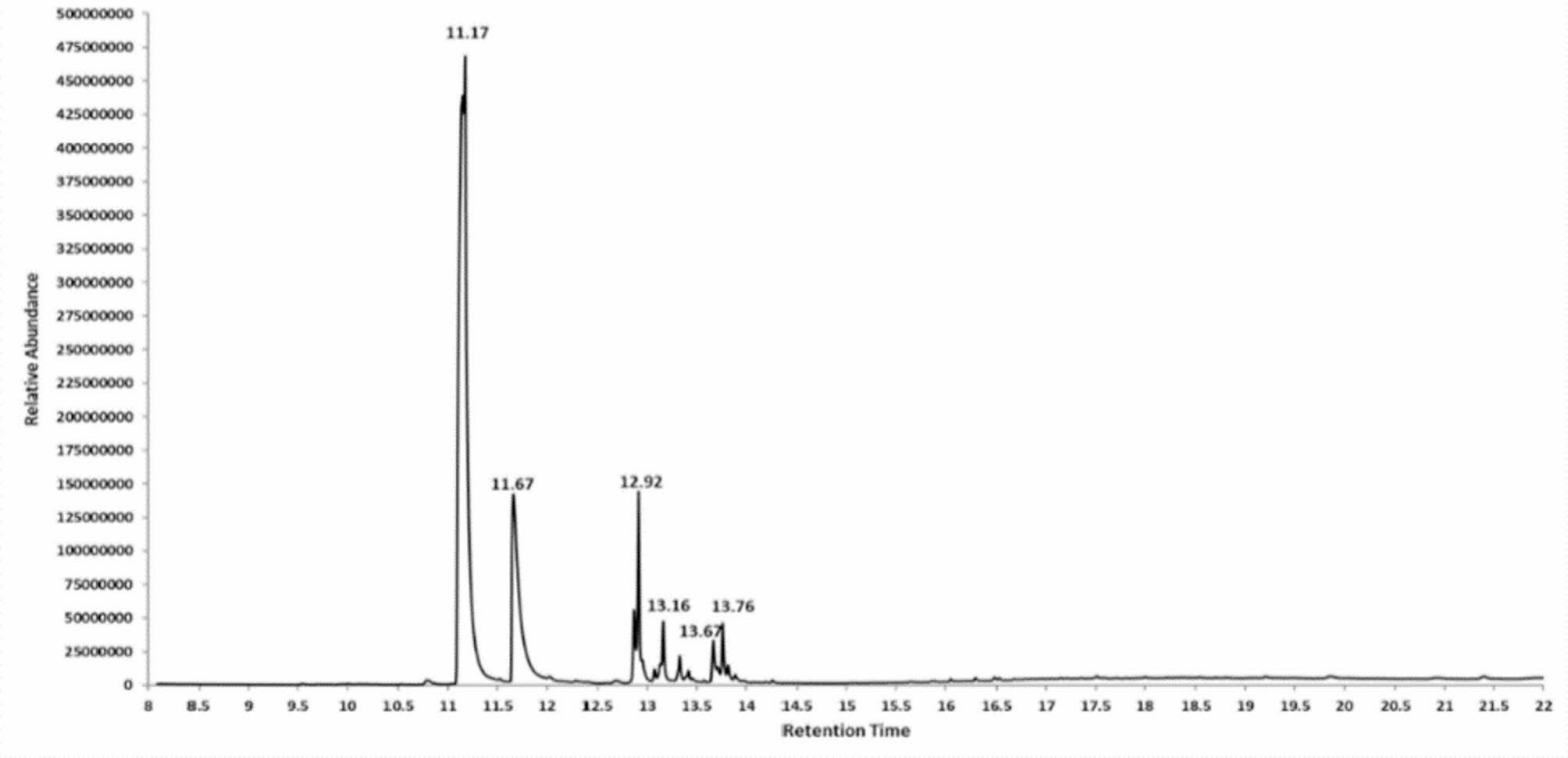
Gas Chromatogram of the ethanolic extract of *Syzygium aromaticum*.

The investigation of the active compounds in the ethanolic extract of *S*. *aromaticum* by GC-MS analysis revealed the presence of eleven major peaks at retention times of 11.17, 11.53, 11.67, 12.03, 12.92, 13.16, 13.33, 13.42, 13.67, 13.76, and 13.89 min. (Fig. 1). The active compounds with their retention time (RT), peak area %, molecular weight, and molecular formula are represented in Table 1. The results showed the presence of eugenol TMS (64.2%), eugenol acetate (18.35%), trans-isoeugenol (0.59%), D-(-)-fructofuranose, pentakis(trimethylsilyl) ether (isomer 1) (7.62%), quininic acid (5TMS) (2.81%), D-galactose, 5TMS derivative (1%), malonic acid, tris-TMS (0.75%), gallic acid, 4TMS derivative (13.67%), glucoside (2.24%), and D-(-)-tagatofuranose, pentakis(trimethylsilyl) ether (isomer 1) (0.48%).

**Table 1:** Qualitative constituents of the ethanolic extract of *Syzygium aromaticum* by GC-MS.

Peaks	Retention Time (min)	% of total	Compound	Molecular Formula	Molecular Weight
1	11.17	63.87	Eugenol TMS	C_13_H_20_O_2_Si	236.38
2	11.53	0.33	Eugenol TMS	C_13_H_20_O_2_Si	236.38
3	11.67	18.35	Eugenol acetate (Phenol, 2-methoxy-4-(2-propenyl)-, acetate)	C_12_H_14_O_3_	206.24
4	12.03	0.59	Trans-Isoeugenol	C_10_H_12_O_2_	164.20
5	12.92	7.62	D-(-)-Fructofuranose, pentakis(trimethylsilyl) ether (isomer 1)	C_21_H_52_O_6_Si_5_	541.06
6	13.16	2.81	Quininic acid (5TMS)	C_22_H_52_O_6_Si_5_	553.07
7	13.33	1.00	D-Galactose, 5TMS derivative	C_21_H_52_O_6_Si_5_	541.06
8	13.42	0.75	Malonic acid, tris-TMS (O, O,O’-Tris-trimethylsilylmalonate)	C_12_H_28_O_4_Si_3_	320.60
9	13.67	1.95	Gallic acid, 4TMS derivative (Benzoic acid, 3,4,5-tris(trimethylsiloxy)-)	C_19_H_38_O_5_Si_4_	458.84
10	13.76	2.24	Glucoside (Glucopyranose, 5TMS derivative)	C_21_H_52_O_6_Si_5_	541.06
11	13.89	0.48	D-(-)-Tagatofuranose, pentakis(trimethylsilyl) ether (isomer 1)	C_21_H_52_O_6_Si_5_	541.06

#### Phytochemical analysis

The results showed that the total alkaloid content (TAC) was 11.53 mg/g _extract_, the total flavonoid content (TFC) was 3.63 mg quercetin /g _extract_, the total phenolic content (TPC) was 139.9 mg gallic /g _extract_, the total tannin content (TTaC) was 27.9 mg/g _extract_, and the total terpenoid content (TTC) was 16.9 mg/g _extract_ (Table 2).

**Table 2:** Quantitative assessment of constituents in extracted materials of *Syzygium aromaticum*.

Constituents	Syzygium aromaticum (mg/g)
**Total Alkaloids (TAC)**	11.53
**Total Flavonoids (TFC)**	3.63
**Total Phenols (TPC)**	139.9
**Total Tannin (TTaC)**	27.9
**Total Terpenoids (TTC)**	16.9

### Acaricidal activity on engorged females

#### External morphological studies

**Fig. 2:**
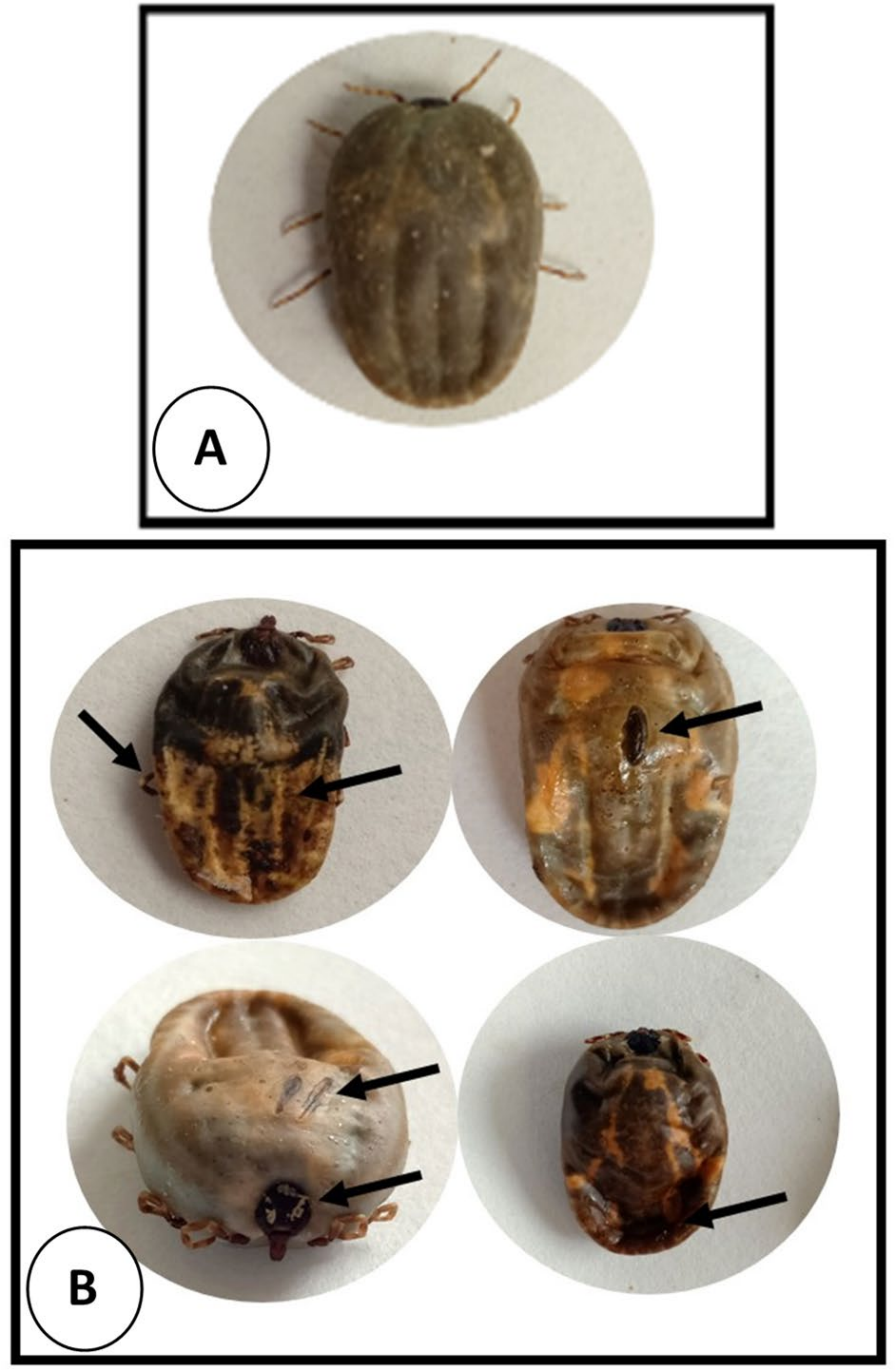
Effect of *Syzygium aromaticum* extract on the morphology of *Hyalomma dromedarii* engorged females. **A** Control female; **B** Treated females.

In the present study, ethanolic extract of *S*. *aromaticum* induced morphological alterations in the engorged female *H*. *dromedarii* (Fig. 2). These alterations included cuticle with great darkness, shrinkage in the body surface with corrugation, stiffened integumental surface, erected legs, hemorrhagic swelling in the treated individuals, and the integument surface appeared with some lesions (Fig. 2). In contrast, the cuticle color of the control individuals appeared normal (greyish white), and there were no signs of hemorrhagic swelling or skin lesions.

#### Mobility and viability

**Fig. 3:**
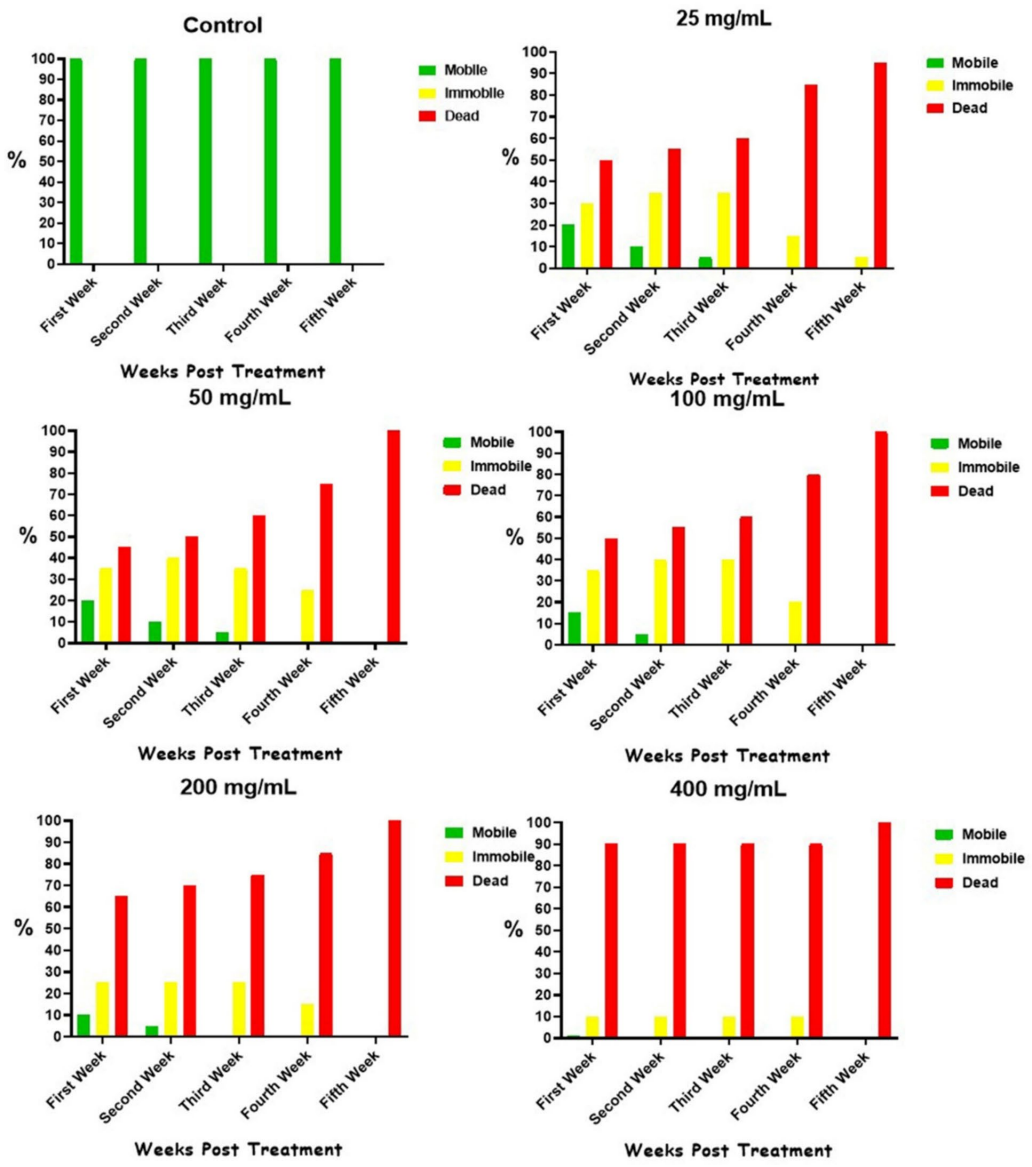
Effect of ethanolic extract of *Syzygium aromaticum* at different concentrations (25, 50, 100, 200, and 400 mg/mL) on the mobility and mortality of *Hyalomma dromedarii* engorged females during five successive weeks (35 days) following treatment.

Generally, the treated ticks showed abnormal and slow movement and did not react to any external stimuli, compared to the control. The control group showed a 100% mobility rate, while that percentage decreased from 20% to 0%, 20% to 0%, 15% to 0%, 10% to 0%, and 0% in the treated groups with 25, 50, 100, 200, and 400 mg/mL, respectively, during the 1st to the 5th week after treatment (Fig. 3). The percentage of immobile treated females decreased, recording 30%-5%, 35%-0%, 35%-0%, 25%-0%, and 10%-0%, respectively, versus 0% in the control group during the examined periods. The percentage of dead ones increased, recording 50%-95%, 45%-100%, 50%-100%, 65%-100%, and 90%-100%, respectively, versus 0% in the control group during the 1st to the 5th week after treatment (Fig. 3).

The calculated LC_50_ of the clove extract was 107.07, 93.6, 100.22, 32.82, and 0.83 mg/mL after the 1st, 2nd, 3rd, 4th, and 5th weeks following treatment, respectively.

**Fig. 4:**
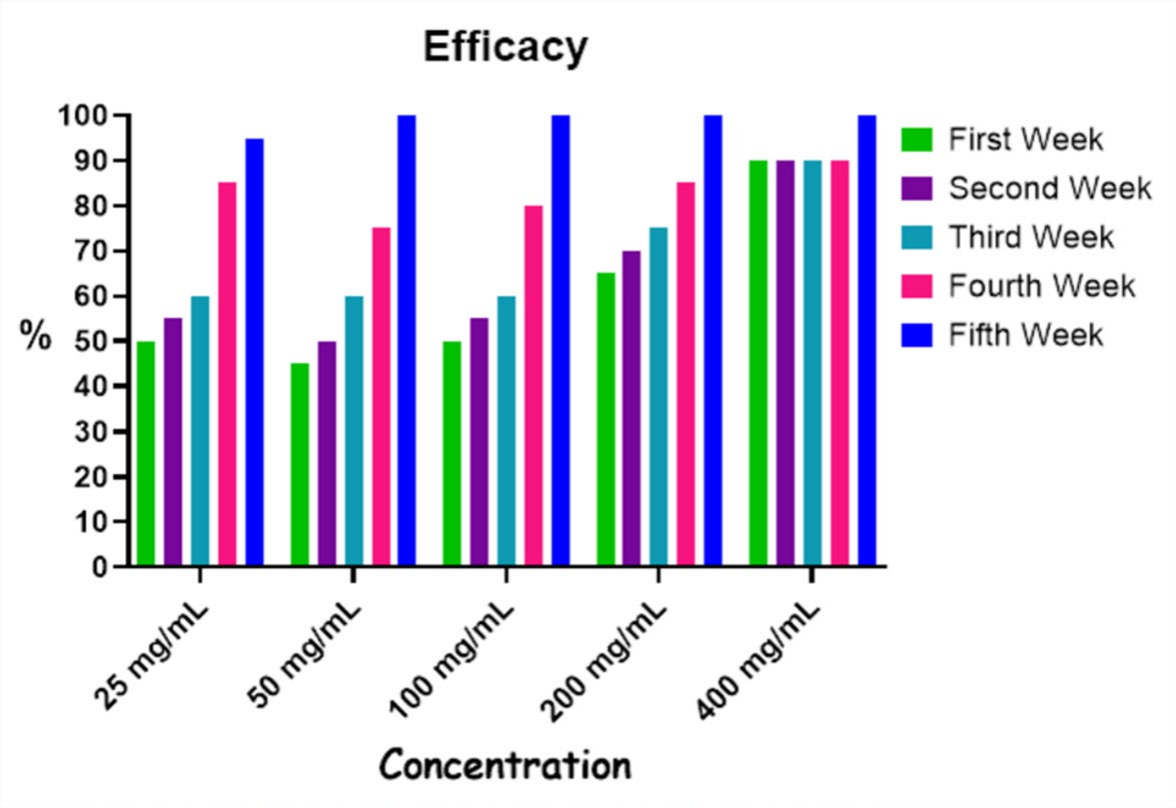
Acaricide efficacy% of ethanolic extract of *Syzygium aromaticum* at different concentrations (25, 50, 100, 200, and 400 mg/mL) against *Hyalomma dromedarii* engorged females.

After the 5th week of treatment, the extract at 25 mg/mL revealed 95% efficacy, and at 50, 100, 200, and 400 mg/mL, it showed 100% efficacy. On the other hand, after the 1st week, this efficacy reached only 45–90% at different concentrations (Fig. 4).

#### Blood digestion

For the control group, the female weights showed a non-significant gradual decrease from zero to the 5th week after feeding, being 853, 808.5, 772, 724.5, 633, and 567 mg, respectively. (Table 3).

Weights of females treated with 25 mg/mL and 50 mg/mL showed a minor digestion from zero to the 1st week (717-452.8 and 735.5–504 mg, respectively). They showed a very highly significant decrease (*p* < 0.0001) for other successive weeks from zero to the 5th week after feeding and treatment (Table 3). Additionally, the great effect of 100, 200, and 400 mg/mL concentrations on the blood digestion appeared in the significant decrease of female weights from zero to the 1st week after feeding and treatment. Females showed a highly significant decrease in their weights from zero to the 5th week after feeding and treatment, from 704.5 mg to 51.5 mg, 784.8 mg to 49.75 mg, and 766.3 mg to 76.11 mg, respectively (Table 3).

**Table 3:** Effect of ethanolic extract of *Syzygium aromaticum* at different concentrations on blood digestion of *Hyalomma dromedarii* engorged females.

Conc. mg/mL	Weight post feeding (mg) Mean ± SE (Range)					
	Zero week	First week	Second week	Third week	Fourth week	Fifth week
Control	853 ± 43.3 (470–1210)	808.5 ± 40.58 (445–1140)	772 ± 38.96 (430–1060)	724.5 ± 37.92 (405–1000)	633 ± 32.81 (365–865)	567 ± 32.81 (290–825)
**25**	**717 ± 45.91** **(385–1145)**	**452.8 ± 62.5** ^**^ **(0-940)**	**226.5 ± 62.65** ^****^ **(0-730)**	**206.5 ± 57.86** ^****^ **(0-695)**	**102.8 ± 43.19** ^****^ **(0-630)**	**40.25 ± 28.59** ^****^ **(0-500)**
**50**	**735.5 ± 49.4** **(345–1125)**	**504 ± 76.14** **(0-960)**	**347.5 ± 82.55** ^***^ **(0-880)**	**323.8 ± 77** ^***^ **(0-820)**	**190.5 ± 68.64** ^****^ **(0-750)**	**40 ± 28.77** ^****^ **(0-515)**
**100**	**704.5 ± 40.71** **(360–1000)**	**374.3 ± 74.58** ^***^ **(0-865)**	**259.8 ± 69.45** ^****^ **(0-795)**	**216 ± 64.26** ^****^ **(0-755)**	**141.5 ± 57.87** ^****^ **(0-690)**	**51.5 ± 35.51** ^****^ **(0-545)**
**200**	**784.8 ± 40.4** **(425–1210)**	**363.8 ± 86.77** ^****^ **(0-1070)**	**215.8 ± 77.19** ^****^ **(0-980)**	**172 ± 69.62** ^****^ **(0-910)**	**89 ± 48.74** ^****^ **(0-650)**	**49.75 ± 34.3** ^****^ **(0-525)**
**400**	**766.3 ± 52.02** **(380–1195)**	**182.6 ± 84.44** ^****^ **(0-1080)**	**136.3 ± 75.15** ^****^ **(0-1040)**	**131.8 ± 72.77** ^****^ **(0-1015)**	**124.7 ± 68.89** ^****^ **(0-965)**	**76.11 ± 53.11** ^****^ **(0-805)**

**: Highly Significant (*p* < 0.01); ***: Very Highly Significant (*p* < 0.001); ****: Very Highly Significant (*p* < 0.0001).

#### Oviposition, hatchability and fertility

The percentage of positive ovipositing females was 100% in the control group. On the other hand, it was 20%, 25%, 30%, 15%, and 10% in the ones treated at 25, 50, 100, 200, and 400 mg/mL concentrations, respectively (Fig. 4). The pre-oviposition period was 28.3 days for the control group and 24.75, 26.2, 25.33, 28, and 25.5 days for the treated groups, respectively (Fig. 4).

**Fig. 5:**
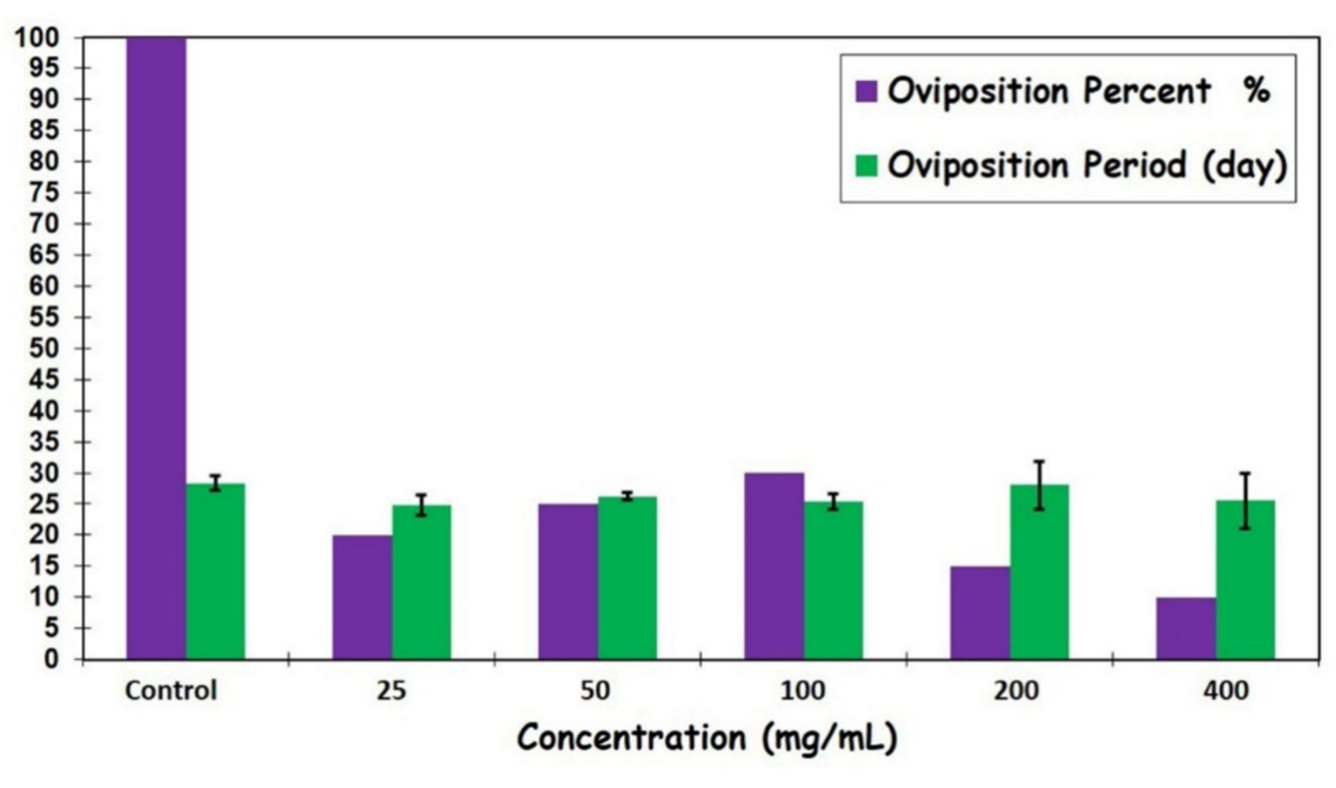
Effect of ethanolic extract of *Syzygium aromaticum* at different concentrations (25, 50, 100, 200, and 400 mg/mL) on the oviposition percent and oviposition period of *Hyalomma dromedarii* engorged females.

The total weight of eggs was 367 mg for the control group, while there was a great decrease in all treated groups (Fig. 5). The control group presented 0.423 fertility, whereas it was (0.057 − 0.027) in the treated groups (Fig. 5). In addition, the inhibition percentage of oviposition reached 93.68% at the highest concentration.

There was a significant increase (*p* < 0.05) in the weight of one egg laid by the females treated with a 25 mg/mL concentration (0.1003 mg) and a very highly significant increase (*p* < 0.001) for the females treated with a 100 mg/mL concentration (0.107 mg), compared with the control (0.0737 mg). The total number of eggs for the control group was 4998, while there was a very highly significant decrease (*p* < 0.0001) in the total number of eggs for all treated groups.

**Fig. 6:**
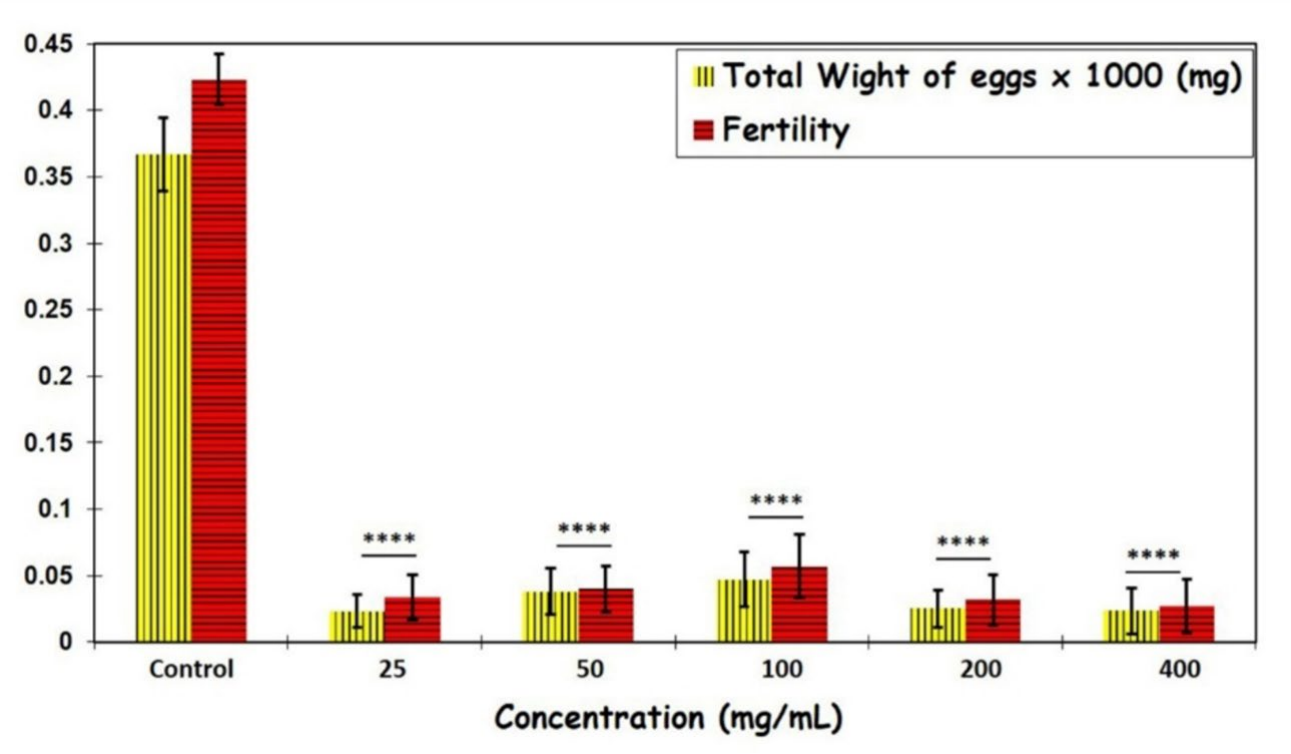
Effect of ethanolic extract of *Syzygium aromaticum* at different concentrations (25, 50, 100, 200, and 400 mg/mL) on the total egg weight and fertility of *Hyalomma dromedarii* engorged females. ****: Very Highly Significant (*p* **< 0.0001).**

The hatching period increased (38.5, 42.75, 35.25, 35.5 and 42 days) for the groups treated with 25, 50, 100, 200 and 400 mg/mL concentrations, respectively, compared with 28.5 days for the control group (Fig. 6). On the other hand, the hatching percentage was 83.75% for the control group and showed a very highly significant decrease (*p* < 0.0001) (39.57%-15.24%) for treated ones (Fig. 6).

The reproductive efficiency (RE) decreased in the treated groups, compared with the control one. According to the reproductive efficiency, the effectiveness of the extract (PE) was 98.09%, 95.32%, 93.73%, 98.65%, and 97.89% at 25, 50, 100, 200, and 400 mg/mL concentrations, respectively.

**Fig. 7:**
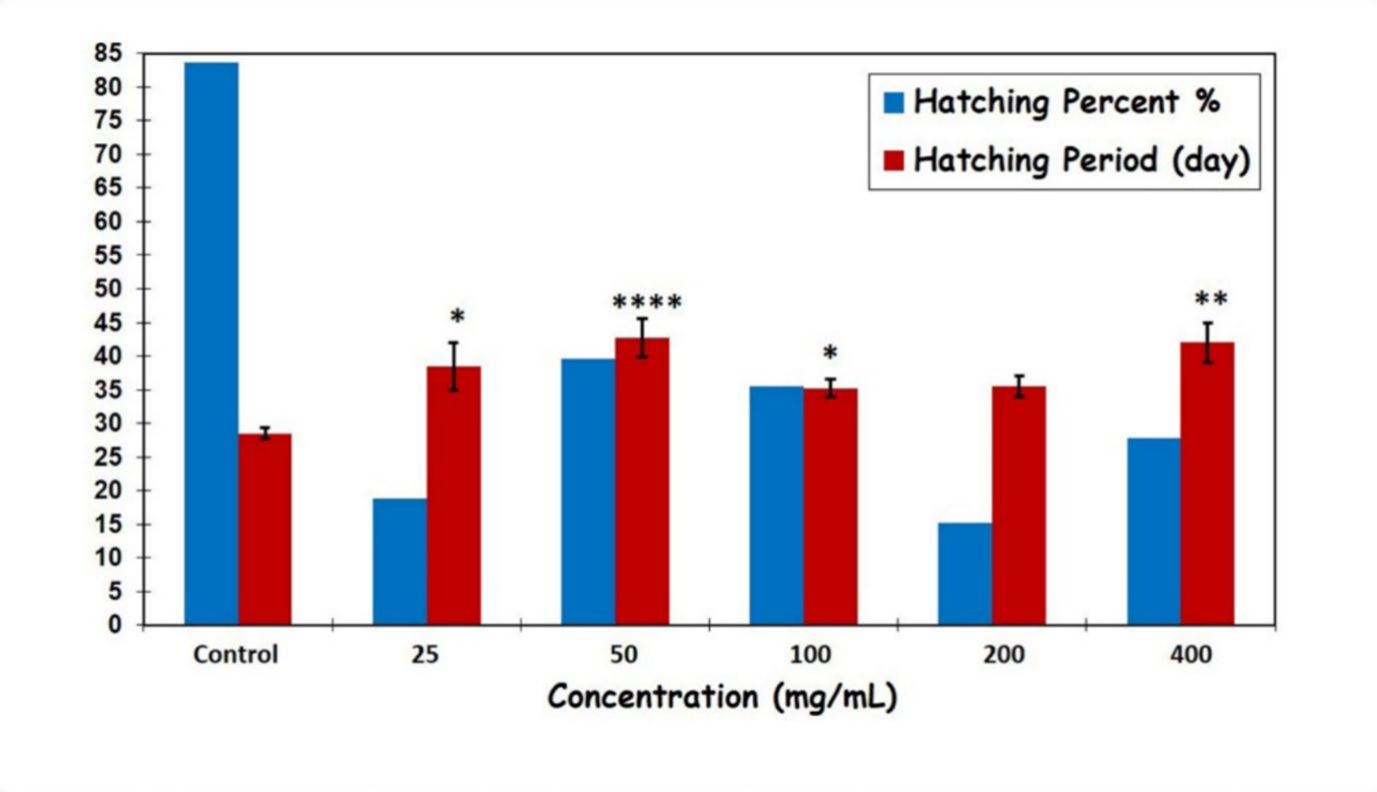
Effect of ethanolic extract of *Syzygium aromaticum* at different concentrations (25, 50, 100, 200, and 400 mg/mL) on the hatching percent and hatching period of *Hyalomma dromedarii* engorged females. *: Significant (*p* < 0.05); **: Highly Significant (*p* < 0.01); ****: Very Highly Significant (*p* **< 0.0001)**.

**Fig. 8:**
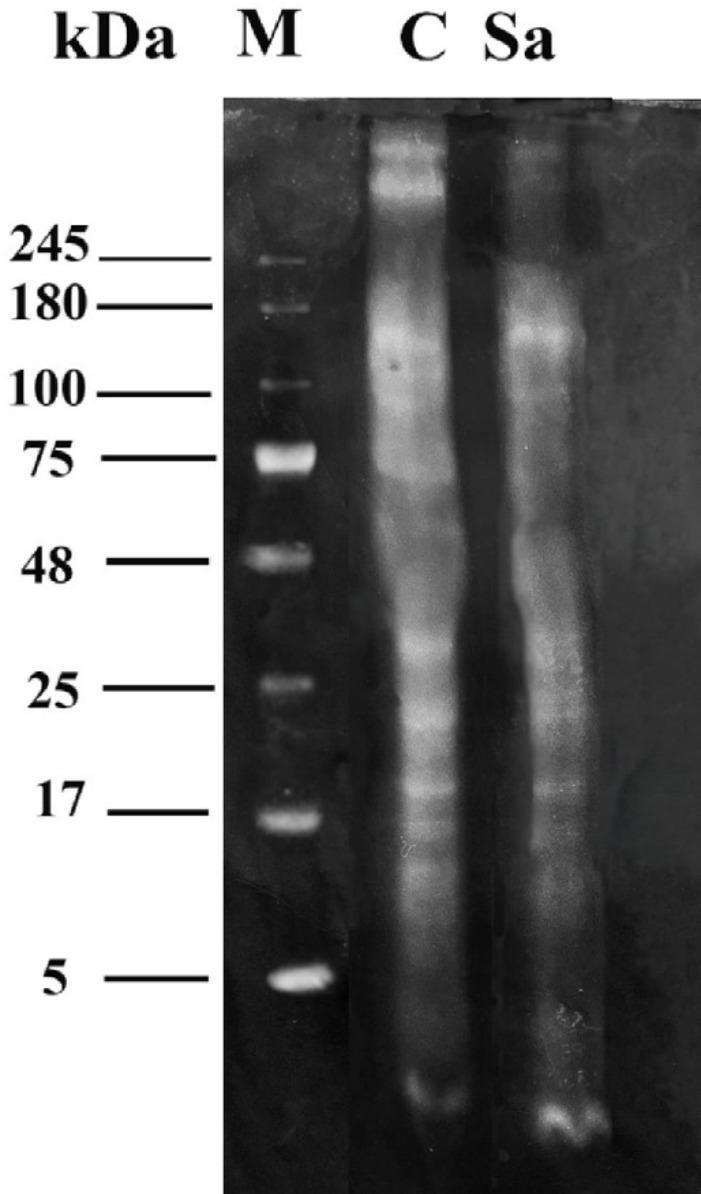
SDS-PAGE electrophoretic profile for total protein of the control and treated female *H*. *dromedarii* with 100 mg/mL *S*. *aromaticum* extract. C= Control individuals: kDa= Kilo Dalton: M= Protein marker: Sa= treated individuals with *S*. *aromaticum* extract.

### Biochemical studies

#### Ovary total protein content

The total protein content of the control ovaries of *H*. *dromedarii* engorged females is 2.703 g/dL. Females treated with 100 mg/mL *S*. *aromaticum* ethanolic extract, showed a highly significant decrease in total protein content in their ovaries, measuring 1.908 g/dL, with *P* < 0.001, indicating a reduction of 26.41% (Table 4).

**Table 4:** Effect of ethanolic extracts of *Syzygium aromaticum* at 100 mg/mL concentration on the total protein content of *Hyalomma dromedarii* engorged female ovary after one week following treatment.

Ovary	Total protein (g/dL) Mean ± SE (Range)
**Control**	2.703 ± 0.009 (2.689–2.721)
***S***. ***aromaticum***	1.908 ± 0.095^***^ (1.728–2.052)

***: Very Highly Significant (*p* < 0.001).

#### Qualitative analysis of ovary protein using sodium dodecyl sulfate polyacrylamide gel electrophoresis (SDS-PAGE)

In the present study, all bands expressed in control individuals showed the highest intensity compared to those expressed in treated ones with *S*. *aromaticum*. The previous observations were clearly demonstrated at 2 bands > 245 kDa, ≈ 140 kDa, ≈ 23 kDa, ≈ 19 kDa, ≈ 17 kDa and < 17 kDa. Treated individuals suffered from the disappearance of 3 bands (≈ 100 kDa, ≈ 75 kDa and ≈ 30 kDa) that were found in the control ones. On the other hand, two high intensity bands in control individuals (molecular size ≈ 90 kDa and ≈ 65 kDa) Fig. 8. SDS-PAGE electrophoretic profile for total protein of the control and treated female *H*. *dromedarii* with 100 mg/mL *S*. *aromaticum* extract. C= Control individuals: kDa= Kilo Dalton: M= Protein marker: Sa= treated individuals with *S*. *aromaticum* extract.

disappeared in treated ones (Fig. 8).

## Discussion

### Plant extract analysis

In the present study, the ethanolic extract yield of *S*. *aromaticum* was 10.6%. Similarly, Alimi et al. ^22^ found 11.3% extract yield of the same clove extract.

The extraction yield of active compounds extracted from plant materials is widely recognized to be influenced by the ratio of water to raw material, making it a crucial factor^60^. Furthermore, the polarity of the extractant plays a key role in increasing the recovery of phenolic and flavonoid compounds^61^. El Mannoubi^60^ demonstrated that the extract yield was 4.67%, 40.92%, and 57.69% when 80% acetone, 80% methanol, and 80% ethanol were employed, respectively. These findings indicate that 80% ethanol is an appropriate solvent for optimizing the extraction yield of Opuntia stricta fruit. The high extraction yields of hydro-alcohol solvents, especially (20:80) hydro-ethanol, can be attributed to their capability to dissolve polar along with non-polar molecules^62^. Also, several other factors impact the extraction yields, including plant variety, ecological characteristics of the harvest area, harvest time, and extraction method^63,64^.

Many studies have investigated the chemical composition of the *S*. *aromaticum* extract and its essential oils^65–69^. Our result is consistent with that of Wagn et al.^70^, who reported that the major compounds in clove alcoholic extract analysis were eugenol and acetyl eugenol. However, they found other compounds such as caryophyllene and humulene, followed by α-farnesene and caryophyllene oxide. These remarkable differences in composition may be due to different extraction methods as well as genetic diversity and agronomic treatments^71^.

It was proven that eugenol has acaricidal activity against several tick species like *Dermacentor nitens*^72^, *Rhipicephalus microplus*^41,72–75^, *R*. *sanguineus*^76–78^, and *R*. *annulatus*^79^. On the other hand, the second component in the present study, eugenol acetate, is a derivative of eugenol that also showed acaricidal activity against *R*. *microplus* with a higher LC₅₀ value than that of non-acetylated eugenol (4.25 mg/mL versus 2.77 mg/mL)^74^. Also, it showed comparable toxicity to benzyl benzoate (the positive control acaricide) against both permethrin-sensitive and resistant *Sarcoptes scabiei* mites with EC₅₀ values: 19.4 mM for sensitive mites and 30.8 mM for resistant mites^40^.

In ticks, the glutathione S transferase (GST) enzyme plays an important role in catalysing detoxification of exogenous and endogenous compounds by increasing the level of glutathione (GSH) and by protecting from reactive oxygen species (ROS) (natural byproducts of metabolism that have toxic effects)^79^. GST was targeted for the development of novel acaricides, as it favors tick survival^80^. Many previous studies revealed that acaricidal resistance in ticks may be due to elevated GST activity^79,81–83^. As Vidhya and Devaraj^84^ found that eugenol caused dose-dependent induction of apoptosis, GSH reduction, and increased reactive oxygen species in human breast cancer MCF-7 cells. Kathiravan et al. ^79^ revealed that these findings could be the causes for its acaricidal property.

As a third component found in the present work, the structure of the fructofuranose derivative D-(-)-tagatofuranose contains a carbohydrate ring with structural homology to the chitin synthase inhibitors (nikkomycin Z, polyoxin A, and polyoxin B)^85^. This hypothesis suggests stopped production of chitin, which leads to the prediction that bioactive compounds (D-(-)-tagatofuranose and D-(-)-fructofuranose) directly affect the integument of the ticks, making them more vulnerable to external conditions, such as heat and light, in addition to weakening their immunity and making it easier for acaricides to pass through their bodies^86^.

The phytochemical results obtained in the present study agree with Tanko et al.^87^, who concluded the presence of flavonoids, resins, glycosides, tannins, saponins, and alkaloids in the ethanolic extract of *S. aromaticum*. In another study done by Upadhyaya et al.^88^, phytochemical screening was carried out on ethanol, chloroform, and distilled water extracts of clove that confirmed the presence of alkaloids, terpenoids, flavonoids, saponins, steroids, and tannins.

The current study showed that phenols were the highest constituent, however, flavonoids were the lowest ones found in clove. Recently, the total phenolic content was 10.53–62.42 mg GAE/g and the total flavonoid content was 1.13–1.47 mg QE/g ^69^. The total phenolic content was 6.23–19.11 mg GAE/g and the total flavonoid content was 5.63–15.32 mg QE/g^89^. Soleimani et al.^90^ suggested that the secondary metabolite profile, which in turn influences the extract activity, could differ between regions due to growth environmental factors and plants’ genotypes.

According to previous studies, the efficiency of plant extracts is considered as the alternative treatment to arthropod control because they are a rich source of the secondary metabolites with active acaricidal activity, including terpenes, stilbenes, coumarins, acids, alcohols, sulphide compounds, tannins, and aldehydes^91,92^. These metabolites present different action mechanisms in controlling ticks, such as inhibition of feeding and chitin synthesis, decreased growth, development and reproduction, and behavioral alterations, without adverse effects to non-target species^93^. Srisanyong et al.^94^ attributed the acaricidal effect of *Artocarpus lakoocha* leaves to the presence of tannin in the leaf layer. Furthermore, flavonoids showed great potential as insect-controlling agents because of their ability to interfere with digestion, growth, development, and reproduction^95^.

### Acaricidal activity on engorged females

Ethanolic extract of *S*. *aromaticum* induced morphological alterations in the engorged female *H*. *dromedarii*, such as great darkness in the cuticle, shrinkage in the body surface with corrugation, erected legs, and hemorrhagic swelling. In accordance with the current study, Habeeb et al.^96^ found that the engorged female *H*. *dromedarii* treated with avermectin and essential oils of *Citrus sinensis* and *C*. *limon* appeared with some morphological alterations, such as a bloated body and splayed legs and they displayed much shallower dorsal ridges than normal healthy ones. Also, de Sousa Martins et al.^97^ reported that the crude extract of *Furcraea foetida* caused lesser hemorrhagic swelling and fewer skin lesions on the engorged female *R*. *microplus* ticks.

In this study, the effects of clove were observed after two days of treatment, particularly at the highest concentrations (200 mg/mL and 400 mg/mL), while all ticks died after 28 days regardless of the concentration used. In agreement with the current study, Alimi et al. ^22^ found that the clove essential oil revealed a potent adulticidal effect at the highest concentration (10 mg/mL), reaching 93.76% mortality, while the ethanolic extract revealed moderate activity of 77.01% on the 15th day after treatment on *H*. *scupense* with LC_50_ value of 3.98 mg/mL. The variance in mortality rates obtained in previous studies can be attributed to differences in susceptibility between tick species^78^.

Many authors studied the adulticidal activity of different plant extracts using the adult immersion test against different ixodid ticks, such as *R*. *microplus*^50,98^, *R*. *decoloratus* and *R*. *pulchellus*^99^, *Amblyomma variegatum*^100^, *H*. *scupense*^53,101^, *H*. *longicornis*^102^, and *H*. *dromedarii*^18,103^. All these plant extracts revealed mortality in adult ticks.

No previous data were detected about the efficacy of *S*. *aromaticum* extract on ticks. Few data were detected about the efficacy of other plant extracts on *Hyalomma* species. Mohamed et al. ^18^ reported the efficacy percentage of different concentrations of *C*. *colocynthi*s fruit extract on *H*. *dromedarii*. The efficacy percentages were 41%, 59%, 82%, 88%, and 94% for the concentrations of 25, 50, 100, 200, and 400 mg/mL, respectively, after the 4th week following treatment.

Gamma-aminobutyric acid (GABA) receptors are crucial targets for controlling ticks^104^. Eugenol demonstrates significant interactions with GABA receptors, which are crucial for nervous system function in arthropods. Clove extracts show the highest potentiation of GABA activity, with eugenol identified as the main contributor to GABAergic activity. This mechanism results in enhanced inhibitory neurotransmission, leading to behavioral suppression and reduced tick mobility^105^.

Previous studies have demonstrated that eugenol and trans-β-caryophyllene are toxic for arthropods^106^. Eugenol from flower buds of clove binds to octopamine receptors, which are specific invertebrate neurotransmitters^106^. The interaction causes increased levels of cyclic adenosine monophosphate (cAMP) and intracellular calcium, disrupting normal cellular processes. Additionally, studies indicate potential interactions with tyramine receptors, which are involved in tick hyperactivity and behavioral responses^107^. Thus, the nerve impulse is blocked, resulting in paralysis of the arthropod^50^. The major compounds of the essential oil of *S*. *aromaticum* are eugenol and trans-β-caryophyllene, which may explain their action on the adult females of *R*. *microplus*^50^. The previous mechanisms may explain the tick paralysis that occurred in treated individuals with the clove extract in the present study.

Ticks digest blood slowly through an intracellular process in their midgut cells^108^. Intestinal digestion of the host blood is an essential process of tick physiology, and its disruption is a limiting factor for pathogen transmission since the tick gut represents the main site for pathogen infection and proliferation^109^.

In the current work, rapid blood digestion occurred in all treated ticks. This assured the morphological alterations found after the examination of treated ticks. There are no previous studies on the effect of *S*. *aromaticum* extract on the blood digestion in *H*. *dromedarii*. Only Mohamed et al. ^18^ investigated the effect of *C*. *colocynthi*s extract from its fruit on the blood digestion of engorged female *H*. *dromedarii*. In accordance with the current study, they reported that the female weights in the control group showed a non-significant, gradual decrease from week zero (835 mg) to the 4th week (624 mg) after feeding, while the weights of females treated with different concentrations showed a significant decrease from week zero to the 4th week after feeding and treatment.

Furthermore, studies on different species reported a significant decrease in weights of treated ticks with greater impact for increasing plant extract concentrations as compared to control, such as for *R*. *microplus*^110–113^, *R*. *sanguineus*^114^, and *R*. *annulatus*^115^.

There is no available data about the effect of clove extract on the oviposition, hatchability, and fertility in *H*. *dromedarii*. However, several authors studied that in other tick species. In agreement with the present study, the clove essential oil also caused a reduction in the oviposition, hatchability, and fertility of *R*. *microplus*^41,50,116^ and *H*. *longicornis*^102^.

Several plant extracts were tested at different concentrations for their ability to reduce fecundity in engorged *Hyalomma* species females, such as *Azadirachta indica* seed extract^117^ and *C. colocynthi*s fruit extract^18^ on *H*. *dromedarii* and *Laurus nobilis*^53^ and *Juniperus communis* and *Origanum majorana*^101^ on *H*. *scupense*.

In arthropods, there are five members in the biogenic amine messenger group: dopamine, tyramine, octopamine, serotonin, and histamine^118^. Octopamine and tyramine play roles in regulating various functions like metabolism, behavior, and physiological functions in arthropods^119^. In addition, octopamine is also implicated in the control of oviposition in insects^120,121^ and ticks^122,123^. The potential neurotoxic and cytotoxic effects of essential oils and their purified components (such as eugenol) on arthropods appear to stem from their ability to bind to tyramine and octopamine receptors, ultimately leading to fatal outcomes^118,119,124^. Toledo et al.^125^ provided evidence that eugenol attaches to and interacts with the lipid-binding surroundings of both anticipated transient receptor potential (TRP) channels and the octopamine receptors in the aphid, *Rhopalosiphum maidis*. The inhibition of fecundity caused by eugenol observed in the present study might be due to its action on the octopamine receptor present in the ticks^79^. Fathy et al.^126^ found that eugenol significantly inhibited the expression of the cyclooxygenase (COX-2) enzyme, thereby decreasing the prostaglandin E2 (PGE2) levels, which in turn is an important signaling molecule involved in the regulation of ovarian maturation and egg laying in invertebrates^127^.

An immunomodulatory protein (RH36) is expressed in the salivary glands of ticks and reaches its peak on the tick engorgement^128^. RH36 gene silencing inhibited tick blood feeding and induced a significant decrease in tick oviposition as it regulates the expression of vitellogenin and ovary cell maturation by modulating the expression of heat shock protein 70 (HSP70), which finally controls tick oviposition. In addition, gene silencing of the HSP70 protein not only inhibited tick blood-sucking and the expression of vitellogenin but also increased the tick death rate^128^. In *R. haemaphysaloides*, the inhibition of RH36 induced a significant decrease in their oviposition rate, ovary weight, and egg-hatching rate^129^. For these reasons, it was hypothesized that the reduction in the oviposition rate and fertility of treated *H*. *dromedarii* females that occurred in the present study may be attributed to the entry of the extract through the mouthparts affecting the expression of RH36 proteins in their salivary glands.

Eugenol can penetrate the cell membrane and change its permeability, both in prokaryotic and eukaryotic cells, facilitating the entry of other compounds into the cytoplasm, which can combine with membrane proteins leading to cell disruption^130–135^. The silico analysis demonstrated the probability and activity of eugenol in affecting the integrity and permeability of the cell membrane through interacting with the phospholipid membrane that delimits the oocytes and yolk granules, causing the histopathological changes in the oocytes of *R*. *sanguineus*^136^. This mechanism of action explains the reduction in the oviposition rate, the total egg numbers, and the hatchability of eggs in the treated females in the present study.

Generally, in published studies, it was observed that clove extract was incorporated in organic solvents without adding adjuvants, which provided stability to the product and adherence to the tegument of the ticks^116^. The acaricidal activity of the clove is attributed to known major components and the resulting synergistic or antagonistic actions^137,138^.

Eugenol accounts for a high proportion of clove components and is the source of the fragrance; it also has the function of killing ticks^72^. Acetylcholine (ACh) and acetylcholinesterase (AChE) exist in ticks as antidotes to excrete toxic substances from the body and are carried out simultaneously in the nervous system^139^. When eugenol enters ticks, it interrupts the action of AChE. In the state where no antidote is used, eugenol continues to react with AChE, causing the hydrolysis step to stop, allowing ACh to continue to be produced, and causing the tick to appear poisoned^140,141^. The acaricidal activity of eugenol has an inseparable relationship with ACh and AChE; this also interferes with the action potential of the tick and has a strong inhibitory effect on AChE activity^142^, causing ACh to continue to act in the body to form a stacking effect and interfere with the operation of the central nervous system and peripheral nervous system^143,144^. A large amount of choline damages the nerve function, causes severe antagonism of the nervous system, and gradually leads to death^145,146^.

### Biochemical studies

*Syzygium aromaticum* extract presented a significant reduction in the total protein content in the ovaries of *H*. *dromedarii*. This reduction revealed an interruption in the physiological regulation mechanism of the rapid developmental stage of the tick ovary, which ensured our biological study that showed a substantial reduction in the fecundity of treated females. The decrease in concentration of ovary protein is predicted to have a direct influence on the ovary and may partially explain the observed reduction in fecundity, fertility, and the delay of oocytes and embryonic development in the treated females as represented by egg number and hatching percent and the latency of oviposition and egg hatching, respectively^147^. The reduction of the total protein in the ovaries deprives the treated female of enough necessary proteins required for the deposition of yolk protein and egg maturation^148^ and embryonic development^149^.

Given that the ovary is a vital tissue of the female reproductive system in ticks and a promising target for controlling them^150^. The SDS-PAGE showed that there were differences in band numbers and molecular weights in the control ovaries and the treated ones. In accordance with our results, the electrophoretic profiles of the ovary of engorged female *H*. *dromedarii* treated with avermectin, *Citrus sinensis* and *C*. *limon* essential oils, and avermectin with each essential oil were investigated by Habeeb et al. ^96^. The ovary homogenates of treated and non-treated ticks revealed the appearance and disappearance of protein bands on the 7th day of injection. Recently, Álvarez-Sánchez et al. ^150^ also reported that the protein gel exhibited contrasting protein band patterns of the ovaries of *R*. *microplus* strains with different levels of resistance to ivermectin.

The profiles of adult proteins and polypeptides, such as vitellin and vitellogenin, were analyzed based on sex, tissue type, and stages of reproductive development^151^. The egg yolk proteins, vitellin and vitellogenin (Vg), a key molecule for oocyte development synthesized in the fat body during blood-feeding, are released into the hemolymph and then taken into the oocytes via the Vg receptor (VgR) in ticks^152^. Their molecular weights ranged from 212 kDa to 35.5 kDa in *H*. *dromedarii*^151^. A study performed by Sattar et al. ^153^ investigated the effect of the clove flower bud extract on the molecular responses of the housefly, *Musca domestica*, and revealed that in the gene expression studies the extract caused elevated stress responses (the detoxification genes CYP12A2, CYP6D2, and CYP6A24 were upregulated) and the reduction of reproductive capacity (the fecundity-related gene Vg-I was downregulated). Furthermore, the molecular docking suggested strong binding affinity of caryophyllene (one of the main components of clove extract) to the survival-related proteins like cytochrome P450 enzymes (which have detoxification and resistance functions) and the vitellogenin domain-containing protein^153^. This interaction with vitellogenin’s lipid-binding domain may impair its function, resulting in suppressed expression and diminished reproductive capacity^153^. The disappearance of the specific protein bands in the present study is directly linked to a reduction in key ovarian proteins. This likely disrupts the vitellogenin synthesis pathway and/or reduction in reproductive capacity genes and detoxification and resistance enzymes which is crucial for yolk information and oocyte development. May be, this reduction explains the mortality and reduced fertility observed in the present biological studies.

Morphologically, the tick fat body is a diffuse tissue closely associated with internal organs, particularly the tracheal system (a network of tubules that extends from the external openings, the spiracles)^154^. It is also located under the epidermis, near the ovaries and the midgut^155,156^. The tick fat body has been reported to be involved in metabolism, energy storage, reproduction (especially synthesis of vitellogenin in fully engorged females), and innate immunity^157–159^. Previously, Schriefer et al.^160^ reported the role of the fat body in ecdysteroid production in *D. variabilis* females. The ecdysteroids injected into the partially fed ticks initiated the expression of the Vg gene, release of Vg into the hemolymph and Vg uptake into developing oocytes^161^, indicating its role in regulating vitellogenesis in ticks^162^. So, the present work supposes that the entry of the extract through the spiracles to the internal organs reached the fat body and affected its function in vitellogenin synthesis. The previous reasons may explain why the clove extract caused a reduction in ovary total protein and the disappearance of ovary protein bands in SDS-PAGE in the present study.

## Conclusions

Given the economic threat of *Hyalomma dromedarii* in Egypt and the harm from current treatments, it was necessary to explore more ecofriendly solutions. This study revealed the great acaricidal activity of *S*. *aromaticum* extract on eggs, larvae, and engorged female *H*. *dromedarii* through affecting their main biological and biochemical parameters, especially ovary total proteins. GC-MS analysis of clove extract observed its chemical composition of eugenol, eugenol acetate, and D-(-)-fructofuranose. It appeared to be rich in alkaloids, flavonoids, phenolic compounds, tannins, and terpenoids using phytochemical analysis. All these compounds had proven to have acaricidal effects. It is recommended that immersion of ticks, especially *H. dromedarii*, in 100 mg/mL *S*. *aromaticum* is a promising green tool to control them, so that the economic burden can be reduced and public health can be better protected. Further works should focus on expanding field studies on a large scale, testing different concentrations of the extract, and monitoring long-term effects to ensure the treatment’s efficacy under field conditions for long-term tick control.

## Data Availability

We are the authors assure that all data and materials support the published claims and comply with field standards. The data are mentioned in the manuscript and will be available after publication.
